# Evaluation of quality of life and socio-emotional impact of oncological treatment among patients with breast cancer

**DOI:** 10.25122/jml-2024-0238

**Published:** 2024-03

**Authors:** Laura Mihaela Mustață, Gheorghe Peltecu, Nicolae Gică, Radu Botezatu, George Iancu, George Dumitru Gheoca, Ruxandra Cigăran, Diana Antonia Iordăchescu

**Affiliations:** 1 Department of Gynecology, Filantropia Clinical Hospital, Bucharest, Romania; 2 Carol Davila University of Medicine and Pharmacy, Bucharest, Romania; 3 Psychology practice, Bucharest, Romania

**Keywords:** breast-conserving surgery, mastectomy, breast reconstruction, quality of life, psychosocial support, resilience, anxiety, depression

## Abstract

Breast cancer is the most frequent cancer in women worldwide. Quality of life (QoL) is significantly affected by both surgical and oncological treatment. The aim of this study was to assess and compare QoL, resilience and depression scores among women who had breast cancer treatment. We assessed 170 patients diagnosed with breast cancer in a non-experimental, descriptive study through anonymized questionnaires from January to March 2024. Patients were invited to fill in the European Organization for Research and Treatment of Cancer Quality of Life Questionnaire, Breast Cancer Module (EORTC QLQ-BR23) questionnaire, the Depression Anxiety Stress Scale, the CD-RISC 10 questionnaire, and the MOS Social Support Survey. Clinical information and demographical data were obtained and statistical analysis was conducted to evaluate factors that affect QoL, resilience and depression scores. QoL was significantly influenced by chemotherapy and surgery. Women with higher resilience scores had lower anxiety and depression scores and reported a better QoL. Women with strong social support and high resilience reported a better QoL during and after breast cancer treatment. The results of our study show that breast cancer surgery and chemotherapy have an important impact on patients’ QoL. Moreover, the results reflect the importance of both medical treatment and social support as resilience-building strategies in managing and improving the QoL of patients.

## INTRODUCTION

Breast cancer remains the most prevalent form of cancer among women worldwide [1,2], and its diagnosis represents a critical event, with potentially traumatic implications. In recent years, the incidence of breast cancer has increased among women under the age of 50 years. As a result, this group has attracted a particularly significant scientific interest. The diagnosis and treatment of breast cancer have improved considerably in recent years, with simple interventions, such as physical activity and psychosocial support, proving to be effective [3,4]. Consequently, the focus is no longer solely on survival but also on preserving the quality of life for breast cancer survivors [5,6].

According to Globocan 2022, breast cancer is the leading cancer among women in Romania, representing 26.8% of all female cancers, with 12,685 cases reported in 2022. In addition, breast cancer is the second most prevalent cancfer in the general population after colorectal cancer, and it has the third highest mortality rate in the country after lung cancer and colorectal cancer, with 3,877 deaths in 2022 [7].

The concept of quality of life (QoL) has gained substantial recognition and importance as a focal point for research and practical application in the fields of health and medicine. The World Health Organization (WHO) defines QOL as “an individual’s perception of their position in life in the context of the culture and value systems in which they live and in relation to their goals, expectations, standards, and concerns” [8]. Additionally, the term health-related QoL (HRQOL) refers to the health-related aspects of QoL, typically indicating the effects of illness and treatment on disability and daily functioning. This concept reflects the perceived health impact on an individual’s ability to lead a fulfilling life [9]. QoL serves as a significant reference point in medical and health research, and understanding QoL is crucial for exploring the consequences of disease and treatment, and for making informed medical decisions across different age groups and cultures [10].

Integrating QoL questionnaires into clinical practice and research endeavors is essential for advancing personalized breast cancer care. By systematically assessing patients’ subjective experiences and incorporating their perspectives into treatment decision-making and academic inquiry, we can optimize therapeutic outcomes, enhance patient satisfaction, and foster holistic approaches to breast cancer management. Continued collaboration between clinicians, researchers, and patients is imperative to refine QoL assessment tools, address unmet supportive care needs, and improve the overall well-being of individuals affected by breast cancer. Historically, the first questionnaires for patients with breast cancer were introduced in the early 1990s. The first studies, which included fewer than 200 patients each from Spain, the Netherlands, and the United States, aimed to evaluate the cross-cultural relevance of standardized questionnaires, which led to the validation of a consistent tool that is now widely used for assessing QoL in breast cancer and many other types of cancer [11,12].

The aim of our study was to investigate how QoL is affected by the diagnosis and treatment of breast cancer. Specifically, the study hypotheses and objectives were as follows:

**Objective 1 (O1):** Assessment of the impact of early diagnosis through screening on the type of surgical intervention and oncological therapy, compared to cases diagnosed at more advanced stages.

**Hypothesis 1A (H1A):** Early diagnosis through screening helps de-escalating the surgical approach (favoring BCS instead of mastectomy) and oncological therapy.

**H1B:** Functional scores (arm and shoulder mobility and pain) are related to type of diagnosis and surgical approach.

**O2:** Examination of the effect of pregnancy/breastfeeding history on the QoL, level of anxiety, resilience, and depression among women diagnosed with breast cancer.

**H2**: History of pregnancy/breastfeeding may yield better scores in QoL, anxiety, resilience and depression.

**O3:** Assess the impact of breast cancer diagnosis and treatment on levels of anxiety, depression, stress, resilience, and QoL in women with breast cancer, analyzing differences based on the type of diagnosis, treatment, and outcome.

**H3A:** There are differences in scores assessing depression, anxiety, stress, resilience and QoL between women who underwent surgical intervention and chemotherapy vs. those who underwent breast-conserving treatment without chemotherapy and other aggressive treatments. Specifically, symptomatic patients (who self-detected a breast lump) have better resilience and depression scores (better social support), and patients who have breast reconstruction (BR) after mastectomy have a better QoL.

**H3B:** There is a positive correlation between levels of anxiety, depression, and stress in women diagnosed with breast cancer.

**O4:** Investigate the relationship between perceived social support and emotional disorders (anxiety, depression, stress) in the context of breast cancer.

**H4:** Social support moderates the relationship between emotional disorders and QoL. Specifically, QoL is positively influenced by perceived levels of social support and negatively influenced by levels of anxiety, depression, and stress in women with cancer.

**O5:** Examine the role of resilience in the relationship between emotional disorders (anxiety, depression, stress) and QoL in women diagnosed with breast cancer.

**H5:** Resilience moderates the relationship between emotional disorders and QoL. Specifically, women with higher resilience experience lower levels of anxiety, depression, stress, and better QoL despite the diagnosis of cancer).

## MATERIAL AND METHODS

### Study design

The study was designed as a non-experimental, descriptive, correlational study in which various hypotheses were tested.

### Procedures and participants

The study was conducted between January and March 2024. Participants were recruited through announcements posted on social media, inviting women diagnosed with breast cancer to take part in a series of scientific studies aimed at assessing psychometrical aspects. Additionally, another cohort of patients was recruited from the Oncology and Surgical Oncology departments of Filantropia Clinical Hospital, Bucharest.

Data were collected online through the Microsoft Forms platform. Before completing the questionnaires, the participants received information regarding the purpose of the study, data collection, and storage methods. Participation was voluntary and anonymous, and all participants provided informed consent.

The study adhered to ethical principles, ensuring data confidentiality and participant anonymity. The instruments and procedures used were noninvasive and designed to avoid causing stress or frustration to participants.

### Instruments

The collected sociodemographic information included age, menopause status, family history, personal history, as well as information about cancer diagnosis and treatment. The participants also completed the following psychological scales:

- The EORTC QLQ-BR23 (European Organization for Research and Treatment of Cancer Quality of Life Questionnaire-Breast Cancer Module) is a specific questionnaire designed to assess the QoL of patients with breast cancer participating in clinical trials or receiving treatment. It is a module of the EORTC QLQ-C30, a widely used instrument for measuring QoL in patients with cancer. The QLQ-BR23 consists of 23 questions that cover specific aspects relevant to patients with breast cancer, including symptoms, body image, sexual functioning, and future perspective. The questionnaire has been extensively validated and has demonstrated reliability and validity in assessing QoL outcomes specific to this population. It is commonly used in clinical trials and research studies to evaluate the effect of different treatments on patients’ well-being and to inform patient-centered care [13].
- For the assessment of anxiety, depression, and stress, we used the 21-item Depression Anxiety Stress Scale (DASS-21) [14]. In this test, respondents rate statements on a 4-point scale based on their experiences over the past week. Subscale scores are converted to *z*-scores for interpretation against normative data. Internal consistency of the scale is high, evidence supports stability over time, and its validity has been established across various populations, including patients with chronic pain and psychiatric conditions.
- To evaluate the resilience of women diagnosed with breast cancer, we used the CD-RISC 10 questionnaire, a shortened version of the original 25-item CD-RISC [15,16]. In this test, each item is rated on a 5-point scale ranging from 'not true at all' to 'true nearly all time', with total scores ranging from 0 to 40 [4]. A preliminary study of its psychometric properties in both general and patient populations have shown adequate internal consistency, test-retest reliability, and convergent and divergent validity.
- The MOS Social Support Survey was created for patients in the Medical Outcomes Study, a two-year study focusing on patients with chronic conditions. It was designed to be comprehensive and distinct from other relevant measures of social support, and it has been validated. The measures of functional social support are reliable and stable over time. The survey consists of four separate social support subscales and an overall functional social support index. A higher score on an individual scale or the overall support index indicates more support [17].

### Statistical analysis

For data analyses was used Python 3.7.4 with the pandas package [18] and matplotlib package [19]. SPSS v.25 for Windows (IBM Corp) was used for data processing and analysis [20]. For graphical representations, we used the seaborn package in Python 3.7.4 [21]. Other statistical tests included one-way analysis of variance (ANOVA), Fisher’s test, and Student’s *t*-test using the SciPy package in Python [22] and SPSS. All statistical tests with a *P* value of ≤0.05 were considered statistically significant.

## RESULTS

### Sample characteristics

Between January and March 2024, we recruited 170 patients diagnosed with breast cancer who received treatment in different cancer centers in Romania (Table 1).

**Table 1:** Demographic and clinical variables of study participants (*n* = 170)

Demographic and clinical variables	Frequency	Percentage
**Age (years)**		
30–40	21	12.4 %
40–50	58	34.1 %
50–65	71	41.8 %
>65	20	11.8 %
Premenopausal	15	9%
Postmenopausal	155	91%
**Age at diagnosis (years)**		
<40	31	18 %
≥40	139	82 %
**Family history of breast cancer or ovarian cancer**		
Yes	54	31.8 %
No	116	68.2 %
**History of pregnancy and lactation**		
At least one pregnancy	149	88%
History of breastfeeding	133	78%
**Oncological treatment**		
Chemotherapy in both neoadjuvant or adjuvant setting	131	77 %
Adjuvant radiotherapy	121	71 %
Endocrine therapy	130	76 %

The demographic data collected included the patients’ age, family history, personal history of pregnancy/lactation, and the type of oncological treatment (Table 1). Given that there is no systematic national screening program in Romania, most patients are diagnosed when they become symptomatic. From the 170 patients included in the study, 118 (69%) self-detected a breast lump, which prompted them to undergo mammography and further investigations. The remaining 52 patients (31%) had a screening mammography despite having no symptoms.

### Testing the study hypotheses

#### H1A: Early diagnosis through screening helps de-escalating the surgical approach (favoring BCS instead of mastectomy) and oncological therapy

Regarding treatment, the vast majority of patients underwent surgical intervention, with only two patients reporting no surgical intervention owing to either ongoing neoadjuvant chemotherapy or advanced-stage disease, metastatic disease, or locally advanced disease. Of the patients who had surgery, 42% had breast-conserving surgery (BCS) and 58% had a mastectomy. Only 28 patients (16%) reported having BR after mastectomy. When asked if they believed that surgical intervention had a negative impact on their QoL, one third of the patients responded affirmatively. In most cases, oncological treatment was associated with the surgical intervention (Table 1).

Among the patients who self-detected a breast lump, 73.4% received chemotherapy, which was similar to the 71.2% of patients diagnosed through screening mammography. Hence, the method of diagnosis did not significantly influence the likelihood of receiving chemotherapy (chi-squared test, *P* = 0.865). Similarly, the frequency of radiotherapy was not influenced by the method of diagnosis, with 71.2% of patients receiving radiotherapy (*P* = 0.858). Although the rate of mastectomy was lower in the screening group (53.8%) compared to the non-screened group (60.2%), the chi-squared test showed no statistical significance difference (*P* = 0.548).

#### H1B: Functional scores (arm and shoulder mobility and pain) are related to type of diagnosis and surgical approach

The type of surgery did not appear to affect arm mobility and pain, regardless of the method of diagnosis (screening vs. symptomatic). Therefore, QoL was not affected from this functional perspective (ANOVA, *P* > 0.05).

#### H2: History of pregnancy/breastfeeding may yield better scores in QoL, anxiety, resilience and depression

History of pregnancy and breastfeeding did not have a positive effect on psychosocial scores, meaning that QoL, depression, and anxiety scores were not better for women who had at least one pregnancy compared to nulliparous women (independent samples *t*-test, *P* > 0.05). Additionally, there were no statistically significant differences between women who breastfed and those who did not (independent samples *t*-test, *P* > 0.05).

#### H3A: There are differences in scores assessing depression, anxiety, stress, resilience and QoL between women who underwent surgical intervention and chemotherapy vs. those who underwent breast-conserving treatment without chemotherapy and other aggressive treatments. Specifically, symptomatic patients (who self-detected a breast lump) have better resilience and depression scores (better social support), and patients who have BR after mastectomy have a better QoL

In our analysis of the psychosocial impact within our cohort, we examined the correlation with the type of diagnosis (self-detection or screening mammogram). Patients who self-detected a breast lump reported having better social support than those who underwent screening mammography (independent samples *t*-test, *P* < 0.01) (Table 2).

**Table 2:** Independent samples *t*-test regarding the difference in social support scales between patients who self-detected a breast lump and patients who underwent screening mammography

	Levene’s test for equality of variances		*t*-test for equality of means						
	*F*	*P* value	*t*	df	*P* value (two-tailed)	Mean difference	Std. error of difference	95% confidence interval of the difference	
								Lower	Upper
Emotional/informational support	0.019	0.891	−2.723	168	0.007	−0.473	0.174	−0.815	−0.130
Tangible support	5.282	0.023	−2.675	168	0.008	−0.524	0.196	−0.911	−0.137
Affectionate support	6.823	0.010	−3.342	168	0.001	−0.643	0.192	−1.023	−0.263
Positive social interaction	0.977	0.324	−3.297	168	0.001	−0.652	0.198	−1.042	−0.262
Overall support score	1.578	0.211	−3.240	168	0.001	−0.544	0.168	−0.876	−0.213

Hypothesizing that BR after mastectomy should lead to a better psychosocial outcome for patients with breast cancer, we compared QoL among patients who underwent mastectomy alone, mastectomy followed by BR, and BCS. Our comparison showed no statistical difference between these three types of surgical treatment regarding functional aspects (pain and shoulder/arm function), depression, anxiety, stress or overall QoL score in our cohort. Moreover, there were no significant differences reported in sexual function regardless of the type of surgery (*P* > 0.05). Another important aspect we investigated was how patients who considered to have a lower QoL due to surgery scored in resilience, stress, and depression scales. The data confirmed an association between lower QoL after surgery, lower resilience, and higher scores in depression and stress (Table 3).

**Table 3:** Independent samples *t*-test regarding the difference in stress, depression, resilience scales between patients who reported that surgery had an emotional impact on their quality of life

	Levene’s test for equality of variances		*t*-test for equality of means						
	*F*	*P* value	*t*	df	*P* value (two-tailed)	Mean difference	Std. error of difference	95% confidence interval of the difference	
								Lower	Upper
Depression	0.264	0.608	2.386	168	0.018	4.104	1.720	0.708	7.500
Stress	0.215	0.644	2.606	168	0.010	4.479	1.719	1.086	7.872
Resilience	0.048	0.827	−2.525	168	0.012	−3.739	1.481	−6.663	−0.816

The validation of this interrelation revealed another important positive correlation between anxiety, depression, and stress scores for all women diagnosed with breast cancer (*P* < 0.01), confirming hypothesis H3B (Table 4).

**Table 4:** Correlation between anxiety, depression and stress in women diagnosed with breast cancer (*n* = 170)

		Anxiety	Depression	Stress
**Anxiety**	***r***	**1**	0.807^*^	0.801^*^
	***P* value (two-tailed)**		**<0.001**	**<0.001**
**Depression**	***r***	0.807^*^	**1**	0.872^*^
	***P* value (two-tailed)**	**<0.001**		**<0.001**
**Stress**	***r***	0.801^*^	0.872^*^	**1**
	***P* value (two-tailed)**	**<0.001**	**<0.001**	

* Correlation is significant at the 0.01 level (two-tailed)

To test the last two hypotheses, H4 and H5, a moderation analysis was conducted using centered variables. The PROCESS SPSS macro was used for data analysis [23]. The interaction effects were statistically insignificant (*P* > 0.05), indicating that the two tested variables, social support and resilience, did not moderate the effect of emotional disorders on QoL. Instead, statistically significant findings indicate that both social support and resilience are predictors of QoL (F (2, 167) = 23.36, *P* < 0.001) (Table 5).

**Table 5:** Summary of linear regression analysis predicting QoL

	*β* coefficient	*t*-statistic	*P* value	95% confidence interval	
				Lower	Upper
Resilience	0.032	4.035	<0.01	0.414	1.202
Social support	0.215	2.708	0.007	1.292	8.242

Additionally, in our study, the resilience score exhibited a significant positive correlation (*P* < 0.01) with the number of friends and close relatives (*r* = 0.29) and with the overall social support score (*r* = 0.50). This suggests that women who have strong social support and who are resilient tend to have a better QoL during and after breast cancer treatment.

**Figure 1:**
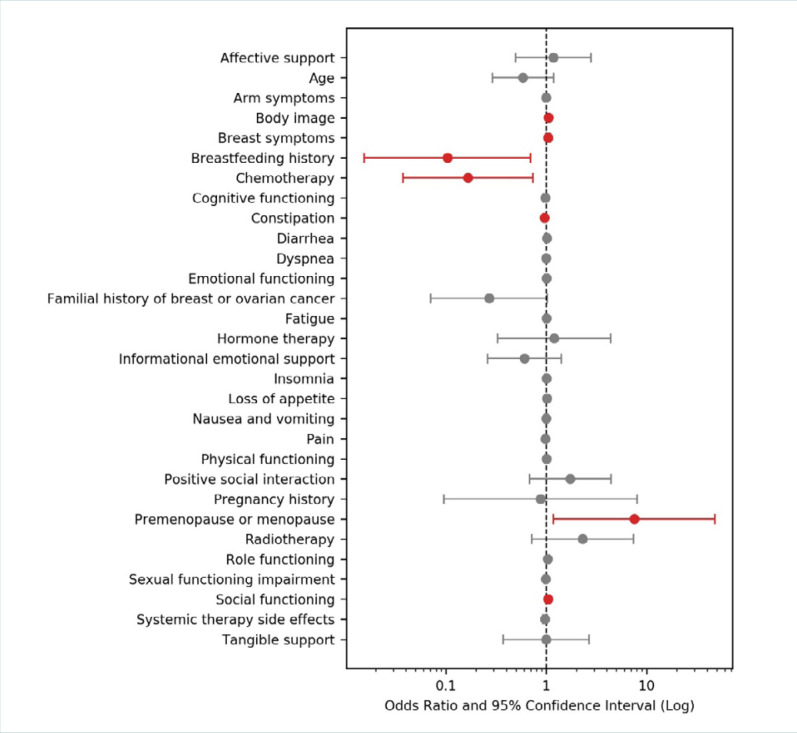
Forest plot representing the results of multinomial logistic regression analysis to identify independent factors that have a positive or negative affect on the QoL of patients with breast cancer

Furthermore, we examined the independent factors that positively or negatively affect the QoL of patients with breast cancer through multinomial logistic regression analysis, using QoL above the median as the dependent variable. In this analysis, approximately 54% of the variability in QoL was explained by the independent variables included in the model. Correct model training was confirmed with a *P* value of <0.001 when compared to a null model with no predictors. Pain and fatigue were the most significant symptoms negatively affecting QoL in our cohort (Figure 1). There was also a strong connection between chemotherapy and lower QoL (OR = 0.166; 95% CI, 0.04–0.73). By contrast, menopausal women reported having better QoL compared to premenopausal women, with positive descriptions of breast appearance, body image, and social interaction.

## DISCUSSION

### Breast cancer overview. Surgical landscape in breast cancer

Breast cancer remains a significant health concern globally, necessitating multidisciplinary approaches for optimal management. Surgical interventions, including BCS and various mastectomy techniques, have pivotal roles in treating breast cancer while considering patients’ QoL outcomes. Research indicates that with appropriate patient selection, BCS and mastectomy offer comparable long-term survival rates. However, differences in cosmetic outcomes and psychosocial adjustment are important considerations. Studies suggest BCS yields superior cosmetic satisfaction and body image perception compared to mastectomy, especially when combined with oncoplastic techniques. Conversely, mastectomy may alleviate anxiety related to cancer recurrence, providing a sense of control over the disease [24–27].

The impact of surgery on QoL extends beyond physical appearance, encompassing functional status, emotional well-being, and social interactions. Postoperative complications, such as lymphedema and chronic pain, can influence patients’ daily activities and overall satisfaction [27]. Moreover, the timing and approach to BR significantly affect QoL outcomes, with immediate reconstruction offering psychological benefits and improved body image perception.

In the context of breast cancer, QoL questionnaires facilitate informed treatment decisions by integrating patients’ perspectives alongside clinical outcomes. Pre-treatment assessments help identify baseline QoL status and anticipate potential treatment-related changes, enabling tailored interventions and shared decision-making. Throughout the treatment continuum, serial QoL assessments inform clinicians about treatment tolerability, symptom burden, and supportive care needs, guiding timely interventions to mitigate adverse effects and enhance patients’ overall experience [28].

Furthermore, QoL questionnaires and phycological assessment have a pivotal role in evaluating the comparative effectiveness of different treatment modalities and supportive interventions. Comparative studies using QoL endpoints provide valuable insights into treatment-related differences in symptom control, functional preservation, and psychosocial well-being, thereby informing evidence-based practice guidelines and optimizing treatment algorithms [29].

### Emotional disorders in breast cancer patients

Stress, anxiety, and depression are intense and complex emotional experiences that can profoundly affect patients diagnosed with breast cancer. Faced with such a diagnosis, these emotions can be heightened and become an integral part of patients’ emotional landscape. Treatment uncertainties, potential side effects of therapies, and concerns for the future contribute to significant stress, anxiety, and, in some cases, depression. Emotional distress in patients with breast cancer is associated with reduced QoL, has a negative effect on medical treatment, and increases mortality risk [30]. Moreover, patients with associated depression report more pain, fatigue, and poorer functioning compared to patients with other types of cancer and are more likely to have suicidal thoughts [31]. Despite its impact on daily functioning, emotional distress is often overlooked and inadequately addressed in patients with breast cancer.

Anxiety is another common psychological symptom encountered in these patients, with prevalence rates ranging between 10% and 30% [32]. This condition can be triggered by the anticipation of negative outcomes and the uncertainty surrounding the future; anxiety may be fueled by concerns regarding recurrence and fear of treatment-related side effects, both during and after treatment [33]. Recent research indicates that anxiety may be even more prevalent than depression, contrary to previous information [34].

Regarding depression, identifying it in patients with breast cancer can be challenging, as depressive symptoms may overlap with physical symptoms associated with the disease or treatment. Among patients with various types of cancer, the prevalence of depression among patients with breast cancer ranks third in the hierarchy of psychological conditions [35]. Depression rates among these patients have been estimated to range between 10% and 30%, varying depending on the studied group, research methodology, and assessment tools used [32]. Studies have found that depression influences women’s therapy, QoL, and personal care capacity, and can affect immunity and survival chances [36]. Many patients with breast cancer experience fatigue, depression, and/or anxiety long after receiving the diagnosis, with these symptoms being associated with greater disability and lower QoL [37].

### Psychological impact, social support and resilience of patients with breast cancer

The highly stressful nature of the disease and its treatment has made research on its psychological effects a major priority. Psychological research has had an essential role in studying the impact of breast cancer on patients’ lives and their families, developing interventions to reduce discomfort and improve QoL, and understanding the possible links between psychological factors and biological processes of the disease [38]. Additionally, research on the psychological aspects of breast cancer provides a valuable framework for studying adaptation to health-related stressors in general.

A recent literature review [39] highlights three trajectories regarding the psychological impact of breast cancer on women: clinical psychological risks, women-specific concerns, and their individual and relational resources, illustrating the complexity of the effects of breast cancer on women under 50 years. It also emphasizes the importance of reflecting on theoretical and psychosocial models to provide support to this group of women while coping with the disease. For these patients, coping skills and emotional recovery resources are associated with multiple variables, which can be assessed through patient characteristics, disease stage, treatment, and environment [40].

How patients cope with their illness and the effects of treatment are central to determining their psychological adaptation to the diagnosis. Cognitive processes, including patients’ thoughts about control over the disease or its role in their lives, are fundamental aspects of this adaptation, and holding relatively optimistic beliefs about future outcomes is associated with better psychological adaptation.

Studies show that coping methods involving avoidance of stressors or negative emotions are associated with poorer psychological adaptation and overall health outcomes. Conversely, approaches that involve confronting stressors and the emotions associated with the diagnosis are associated with positive psychological outcomes [41]. Several studies have shown that the manner in which patients manage stress can influence the level of discomfort associated with breast cancer, with the lowest levels reported among those who adopt an active, problem-solving-oriented approach and have a sense of control over their situation [42].

Social support is essential in the care of patients with cancer, yet it is often underutilized owing to the patients’ lack of initiative in seeking help and emotional support or to the inability of their family and friends to adequately respond to their needs [43]. Moreover, social support has a crucial role in positive adaptation to breast cancer. A study by Holland and Holahan [44] revealed a positive correlation between perceived social support and positive adaptation to the illness. Specifically, participants who received higher levels of social support exhibited better adaptation and higher psychological well-being following a breast cancer diagnosis and treatment.

In terms of treatment options, psychological interventions such as cognitive-behavioral therapy, supportive-expressive group therapy, and yoga have shown positive effects in patients with breast cancer, whereas classical pharmacotherapy, such as antidepressants, should be reserved for cases of diagnosed depression [45]. Preliminary evidence suggests that psychosocial interventions for patients with breast cancer could have beneficial physiological effects, including decreased cortisol levels, improved immune function, and increased survival [46].

### Mastectomy vs. BCS and their implication in QoL

Our study aimed to explore the impact of breast cancer diagnosis and treatment on patients’ QoL. Contrary to our initial expectations, we did not find a significant difference in QoL based on the diagnostic method (mammographic screening or self-detection of a breast lump) and the subsequent oncological treatment received. Although mastectomy rates were slightly lower among patients diagnosed through screening, the difference was not statistically significant.

An important finding is that approximately one-third of patients reported that surgical intervention had a negative impact on their QoL. This indicates that despite the potential benefits of early detection and conservative breast approaches, surgical intervention can still have a significant impact on the QoL of patients.

Studies suggest that breast cancer screening has a critical role in early detection and improved outcomes for patients. Since the introduction of screening with mammography in the late 1960s and early 1970s [47,48], advancements in breast cancer therapy and treatment strategies have been substantial. Screening enables the detection of abnormalities such as microcalcifications and masses before they become palpable, facilitating early intervention and potentially curative treatment [49–51].

Both mastectomy and BCS are effective treatments for breast cancer, and the choice between the two depends on various factors, including tumor size and location, patient preferences, cosmetic considerations, and oncological outcomes. Additionally, both procedures can be followed by BR to restore breast appearance, underscoring the importance of patient-centered care in breast cancer treatment and decision-making. Studies on long-term QoL have shown that both BCS with radiotherapy and mastectomy with BR and radiotherapy yield similar scores in terms of well-being and QoL. However, women undergoing BCS report a positive and meaningful effect on sexual well-being compared to those who undergo mastectomy with BR. Therefore, the decision-making process should definitely include these aspects for patients with early breast cancer [52].

Our study also examined the relationship between pregnancy/breastfeeding history and women’s psychosocial scores. No positive correlation was observed between these factors and psychosocial scores. Although breastfeeding is associated with numerous physical health benefits for both mother and child, including reducing the risk of breast cancer [39], its effect on psychosocial well-being may be less significant than previously thought. These findings underscore the need for further research to better understand the relationship between pregnancy/breastfeeding history and women’s psychosocial status. Similarly, the type of surgical approach may not have a substantial effect on functional outcomes and QoL. However, more research is needed to explore these relationships comprehensively and identify other potential factors that may influence patient outcomes.

Rosenberg *et al*. found that QOL and psychosocial well-being in young patients with breast cancer tend to improve over time. However, extensive surgery and aggressive oncological treatment have a negative effect on sexual well-being, self-esteem, and anxiety levels [29]. In our study, patients who self-detected a breast lump reported better social support than patients diagnosed through screening, and menopausal patients reported better QOL than premenopausal patients.

Although BCS followed by radiotherapy gained significant popularity after 1990, supported by studies demonstrating comparable results to mastectomy [53,54], including a meta-analysis of more than 1,500,000 patients that showed improved overall survival after BCS vs. mastectomy [55], surgical preferences are changing both among patients and medical professionals [24]. Patients tend to increasingly favor mastectomy with BR, either immediate or delayed, and also contralateral prophylactic mastectomy, especially younger patients diagnosed before the age of 40 [25,56–58].

Overall, although there is no definitive answer as to whether QoL is universally better after mastectomy or BCS, studies suggest that the latter is associated with more favorable QoL outcomes, particularly in terms of body image and self-esteem. However, the decision between mastectomy and BCS should be individualized and based on factors such as tumor characteristics, patient preferences, and overall treatment goals, with careful consideration of potential QoL implications [53].

### Social and functional dimensions of QoL

QoL questionnaires have an important role in tailoring treatment strategies for patients with breast cancer. Although functional scales are useful for evaluating patients’ ability to perform daily activities, such as self-care, work and leisure activities, as well as their independence and mobility, there is also an important social dimension to these instruments. Social dimensions may include questions about the level of support from family and friends, satisfaction with social activities, and perceived social support networks. Therefore, tailored support services should be included to address specific social and functional needs [59–61].

### Integrating QoL assessments into clinical practice

The comparison of QoL outcomes between mastectomy and BCS in patients with breast cancer has been the subject of numerous studies. Although findings vary somewhat depending on study design, patient population, and follow-up duration, several trends have emerged. Many studies have found that BCS generally leads to better body image and higher self-esteem compared to mastectomy. This is likely because BCS preserves much of the breast, resulting in less noticeable physical changes compared to mastectomy [62,63]. Our findings suggest that BR after mastectomy may not necessarily lead to better psychosocial outcomes for patients with breast cancer. We compared QoL among patients who underwent mastectomy, those who had mastectomy and BR, and patients who underwent BCS. We found no statistical difference between these three types of surgical treatments regarding functional aspects, such as pain and shoulder/arm function, depression, anxiety, stress, or overall QoL score in our cohort. Additionally, we found no significant differences in sexual function regardless of the type of surgery performed.

Studies examining psychological and emotional states after mastectomy vs. BCS have yielded mixed results. Although some studies have reported similar levels of psychological distress between the two groups, others have found that women who undergo BCS experience less anxiety and depression.

Overall treatment satisfaction may be higher among women who undergo BCS compared to mastectomy, as BCS allows for preservation of the breast and often results in improved cosmetic outcomes. In terms of physical functioning and symptom burden, studies have found similar outcomes between mastectomy and BCS. However, women who undergo mastectomy may experience more physical discomfort and limitations immediately following surgery, which can affect short-term QoL [64].

Long-term survivorship outcomes, including QoL, may be influenced by factors such as adjuvant treatments (e.g., radiation therapy or chemotherapy), BR, and psychosocial support, in addition to the type of surgical intervention [60].

Oncological treatment for breast cancer, which includes chemotherapy, radiation therapy, hormone therapy, and targeted therapy, has a crucial role in improving survival outcomes. However, these treatments can significant affect the QoL of patients with breast cancer. Chemotherapy, for instance, is associated with side effects such as fatigue, nausea, hair loss, and cognitive impairment, affecting physical, emotional, and social well-being. Similarly, radiation therapy may cause skin irritation, fatigue, and long-term effects such as lymphedema and cardiac toxicity. Hormone therapy and targeted therapy, although generally better tolerated, can also lead to side effects such as hot flashes, joint pain, and mood changes. The cumulative burden of these treatment-related side effects can affect patients’ daily functioning, psychological well-being, and overall QoL.

Chemotherapy is a significant factor in assessing the QoL of patients with breast cancer, as it is often associated with various physical and psychological side effects that can affect well-being during and after treatment. Physical side effects, such as fatigue, nausea, vomiting, hair loss, changes in appetite, neuropathy, and decreased immune function, can affect patients’ ability to carry out daily activities, work, and socialize, thereby impacting their overall QoL [65,66]. Chemotherapy can also have psychological effects, including anxiety, depression, fear of treatment, and concerns about treatment effectiveness and long-term side effects. These psychological factors can contribute to decreased QoL and may require additional support and interventions [67].

An important aspect analyzed in our study was the relationship between the patients’ perceived lower QoL due to surgery and their scores on resilience, stress and depression scales. The data confirmed the association between lower QoL after surgery, lower resilience, and higher depression and stress scores. This finding highlights the importance of addressing psychosocial well-being alongside surgical treatment. Patients who reported lower QoL after surgery may benefit from additional support to enhance their resilience and alleviate depression and stress symptoms. By recognizing and addressing these psychological aspects, healthcare providers can better support patients throughout their treatment journey, ultimately improving their overall well-being and QoL. According to a recent study, cognitive behavioral stress management may enhance emotional QoL during the first year of breast cancer treatment for women reporting low optimism [68].

The results of our study highlight two significant predictors of QoL among patients with breast cancer: social support and resilience. Resilience scores showed a positive correlation with the number of friends and close relatives and with the overall social support score. These findings suggest that women with robust social support networks and higher levels of resilience tend to have better QoL during and after breast cancer treatment. In light of these results, it becomes evident that psychosocial interventions aimed at enhancing social support and resilience could significantly improve the QoL of these patients.

A recent study that explored the role of social support in the relationship between resilience, hope and QoL in patients with breast cancer concluded that all three variables had a significant effect on health-related QoL [69]. Other studies also reinforce the importance of social support in improving the QoL of patients, as it can influence the process of adaptation to cancer diagnosis and treatment [70].

Our results highlighted several important aspects related to QoL among patients with breast cancer. The first aspect is related to the symptoms of pain and fatigue, which were identified as the most significant negative factors affecting the QoL of these women. These findings underscore the profound impact of these symptoms on the daily life and overall emotional state of patients with breast cancer. We also identified a strong link between chemotherapy and reduced QoL, suggesting that the side effects of chemotherapy, such as fatigue, nausea and other adverse symptoms, may have a significant effect on patients’ QoL. It is important to recognize these side effects and provide patients with adequate support to manage them.

Some patients with breast cancer undergoing chemotherapy may experience cognitive changes commonly referred to as ‘chemo brain’ or ‘chemo fog’. These cognitive impairments can affect memory, concentration, and executive function, impacting work, relationships, and overall QoL [71,72]. Chemotherapy can be emotionally challenging for these, leading to feelings of uncertainty and emotional distress. Coping with the demands of treatment, fear of recurrence, and adjusting to changes in physical appearance can all affect emotional well-being and QoL [73–75]. Although chemotherapy is often effective in treating breast cancer, some patients may experience long-term or late effects of treatment that can affect QoL, such as cardiac toxicity, bone health issues, and increased risk of secondary cancers. Monitoring and managing these long-term effects are essential strategies for optimizing the QoL of breast cancer survivors [76,77]. Providing comprehensive supportive care, symptom management, and psychosocial support can help mitigate the negative effects of chemotherapy on QoL and improve overall patient outcomes.

Although effective in improving survival rates, radiation therapy can affect various aspects of a patient’s QoL. Side effects such as skin irritation, fatigue, and changes in breast appearance may occur during or after treatment, influencing physical well-being and body image. Additionally, radiation therapy can also lead to emotional distress, anxiety about treatment efficacy, and concerns about long-term side effects, affecting psychological well-being and overall QoL [78,79].

An interesting observation of our study is that postmenopausal women reported better QoL compared to premenopausal women. This finding suggests that despite the challenges associated with menopause and breast cancer treatment, postmenopausal women may experience an improvement in their overall health and well-being. Moreover, these women described their breast appearance, body image, and social interactions positively, which shows a better perception of their own health and self-confidence.

Other research suggests that BCS may have less of an impact on sexual functioning compared to mastectomy, as it often preserves more breast tissue and may be associated with fewer body image concerns related to sexual intimacy [64,80,81].

### Future directions

Future research should aim to tailor breast cancer treatment and strategies to improve the QoL of patients, reduce the rate of extensive surgical treatment with early detection of breast cancer, reduce the physical and psychological effects of oncological treatment, raise awareness among general population on the importance of screening, identify strategies to increase resilience among women diagnosed with breast cancer, identify risk and protective factors associated with stress, anxiety, and depression in women with breast cancer, evaluate the impact of breast cancer diagnosis and treatment on mental health and emotional well-being, and identify effective interventions for reducing emotional disorders and improving QoL among women with breast cancer.

In addition, our study highlights several aspects of breast cancer management that need to be further explored and better understood to improve patient care and treatment. The medical and psychosocial aspects analyzed in our study raise important issues regarding the improvement of QoL for patients with breast cancer, suggesting several future directions for research and interventions:

- Education for breast cancer screening in Eastern European countries with no active screening policy. Greater efforts are needed to educate the population about the importance of screening and early detection of breast cancer. Implementing a national screening program is necessary to increase the chances of early diagnosis and effective treatment.
- Balanced treatment decisions. There is a need for a more balanced approach to treatment decisions, taking into account both the physical and emotional aspects of the disease. More education and counseling could help patients make more informed and appropriate decisions regarding their treatment.
- Greater cohesion and standardization in breast cancer treatment in Eastern European countries with no active screening policy. Adapting and individualizing treatment for each patient is essential for achieving the best results.

### Challenges and limitations

It is important to mention the challenges and limitations of our study. One of the most important challenges we encountered during this study was the social aspect of breast cancer, more specifically the shame and stigma experienced by these patients following their diagnosis, the difficulty of talking about body image, sexual dysfunctions, or other issues resulting from the diagnosis. Another major challenge was that patients in Romania are not educated for medical and psychological screening, as there is no national screening program. Consequently, they tend to request radical surgeries or treatments regardless of the subsequent psychological impact, leading to a strong socio-emotional impact that is difficult to manage. Additionally, there is a lack of unity in treatment and medical recommendations despite the existence of national guidelines. Treatment needs to be adapted and individualized in multidisciplinary meetings; however, there is significant heterogeneity across different cancer centers.

The limitations of our study include the small sample size compared to other studies in the literature, the short period of time during which the questionnaires were administered, and incomplete medical data given that the study is based on patient responses, which may lead to social desirability bias. Additionally, the heterogeneity of the sample, recruited from multiple treatment centers in different cities, may influence the results. We do not have data on the time from diagnosis to completion of treatment, and the online questionnaire was completed only by those who wished to participate, limiting our access to more data or to patients with potentially more significant emotional disorders.

Despite these limitations, our study has notable strengths. We used internationally standardized questionnaires, which enabled us to analyze and correlate data from patient cohorts from different breast cancer care centers. Furthermore, this study contributes to the existing knowledge about emotional distress, social support, and resilience associated with breast cancer, especially among women in Romania. The results are expected to inform the development of medical programs that emphasize the importance of screening and QoL in the treatment of patients with breast cancer, as well as appropriate methods of therapeutic intervention. Organizing support groups for these patients and developing medical and psychotherapeutic/psychoeducational intervention programs that focus on developing strategies to cope with emotional disturbances can have positive effects on their QoL.

## CONCLUSION

In conclusion, our results demonstrate that breast cancer surgery can have a substantial impact on patients’ QoL, with pain and fatigue being the most significant negative symptoms reported. Social support and resilience levels have been identified as crucial factors in determining QoL after breast cancer diagnosis and treatment. Women with strong social support and high resilience tend to have a better QoL during and after treatment. Additionally, our findings show a correlation between chemotherapy and lower QoL, and menopausal women reported a better QoL compared to premenopausal women. These findings highlight the importance of not only medical treatment but also social support and resilience-building strategies in managing and improving the QoL of patients with breast cancer.

## Data Availability

Further data is available from the corresponding author on reasonable request.

## References

[1] DeSantis C, Siegel R, Bandi P, Jemal A (2011). Breast cancer statistics, 2011. CA Cancer J Clin.

[2] Momenimovahed Z, Salehiniya H (2019). Epidemiological characteristics of and risk factors for breast cancer in the world. Breast Cancer (Dove Med Press).

[3] Mokhatri-Hesari P, Montazeri A (2020). Health-related quality of life in breast cancer patients: review of reviews from 2008 to 2018. Health Qual Life Outcomes.

[4] van Leeuwen M, Husson O, Alberti P, Arraras JI, Chinot OL, Costantini A (2018). Understanding the quality of life (QOL) issues in survivors of cancer: towards the development of an EORTC QOL cancer survivorship questionnaire. Health Qual Life Outcomes.

[5] Bottomley A, Reijneveld JC, Koller M, Flechtner H, Tomaszewski KA, Greimel E, 5th EORTC Quality of Life in Cancer Clinical Trials Conference Faculty (2019). Current state of quality of life and patient-reported outcomes research. Eur J Cancer.

[6] Fayers PM, Machin D (2016). Quality of life: the assessment, analysis and reporting of patient-reported outcomes.

[7] Globocan (2022). https://gco.iarc.who.int/media/globocan/factsheets/populations/642-romania-fact-sheet.pdf.

[8] (1995). The World Health Organization quality of life assessment (WHOQOL): Position paper from the World Health Organization. Soc Sci Med.

[9] Mayo N (2015). Dictionary of Quality of Life and Health Outcomes Measurement.

[10] Haraldstad K, Wahl A, Andenæs R, Andersen JR, Andersen MH, Beisland E (2019). A systematic review of quality-of-life research in medicine and health sciences. Qual Life Res.

[11] Sprangers MA, Groenvold M, Arraras JI, Franklin J, te Velde A, Muller M (1996). The European Organization for Research and Treatment of Cancer breast cancer-specific quality-of-life questionnaire module: first results from a three-country field study. J Clin Oncol.

[12] Aaronson NK, Ahmedzai S, Bergman B, Bullinger M, Cull A, Duez NJ (1993). The European Organization for Research and Treatment of Cancer QLQ-C30: a quality-of-life instrument for use in international clinical trials in oncology. J Natl Cancer Inst.

[13] Bjelic-Radisic V, Cardoso F, Cameron D, Brain E, Kuljanic K, da Costa RA (2020). An international update of the EORTC questionnaire for assessing quality of life in breast cancer patients: EORTC QLQ-BR45. Ann Oncol.

[14] Lovibond PF, Lovibond SH (1995). The structure of negative emotional states: comparison of the Depression Anxiety Stress Scales (DASS) with the Beck Depression and Anxiety Inventories. Behav Res Ther.

[15] Connor KM, Davidson JR (2003). Development of a new resilience scale: the Connor-Davidson Resilience Scale (CD-RISC). Depress Anxiety.

[16] Campbell-Sills L, Stein MB (2007). Psychometric analysis and refinement of the Connor-Davidson Resilience Scale (CD-RISC): Validation of a 10-item measure of resilience. J Trauma Stress.

[17] Sherbourne CD, Stewart AL (1991). The MOS social support survey. Soc Sci Med.

[18] Lightner L, Brywczynski J, McKinney J, Slovis CM Data Structures for Statistical Computing in Python.

[19] Hunter JD (2007). Matplotlib: A 2D Graphics Environment. Comput Sci Eng.

[20] IBM Corp (2017). Released IBM SPSS Statistics for Windows, Version 25.0.

[21] Waskom ML (2021). Seaborn: statistical data visualization. J Open Source Softw.

[22] Virtanen P, Gommers R, Oliphant TE, Haberland M, Reddy T, Cournapeau D (2020). SciPy 10: fundamental algorithms for scientific computing in Python. Nat Methods.

[23] Hayes AF (2022). Introduction to mediation, moderation, and conditional process analysis: A regression-based approach.

[24] Lazow SP, Riba L, Alapati A, James TA (2019). Comparison of breast-conserving therapy vs mastectomy in women under age 40: National trends and potential survival implications. Breast J.

[25] Noguchi M, Yokoi-Noguchi M, Ohno Y, Morioka E, Nakano Y, Kosaka T (2016). Oncoplastic breast conserving surgery: Volume replacement vs. volume displacement. Eur J Surg Oncol.

[26] Lyons JM, Chu QD, Hsieh MC, Wu XC (2021). Breast-Conserving Therapy vs Mastectomy for Early-Stage Breast Cancer: Should We Re-Evaluate the Current Treatment Paradigm? Reply to Jatoi. J Am Coll Surg.

[27] Hanson SE, Lei X, Roubaud MS, DeSnyder SM, Caudle AS, Shaitelman SF (2022). Long-term Quality of Life in Patients With Breast Cancer After Breast Conservation vs Mastectomy and Reconstruction. JAMA Surg.

[28] Huynh V, Yang J, Bronsert M, Ludwigson A, Ahrendt G, Kim S (2021). Choosing Between Mastectomy and Breast-Conserving Therapy: Is Patient Distress an Influencing Factor?. Ann Surg Oncol.

[29] Rosenberg SM, Dominici LS, Gelber S, Poorvu PD, Ruddy KJ, Wong JS (2020). Association of Breast Cancer Surgery With Quality of Life and Psychosocial Well-being in Young Breast Cancer Survivors. JAMA Surg.

[30] Linden W, Vodermaier A, Mackenzie R, Greig D (2012). Anxiety and depression after cancer diagnosis: prevalence rates by cancer type, gender, and age. J Affect Disord.

[31] Walker J, Hansen CH, Martin P, Symeonides S, Ramessur R, Murray G (2014). Prevalence, associations, and adequacy of treatment of major depression in patients with cancer: a cross-sectional analysis of routinely collected clinical data. Lancet Psychiatry.

[32] Ng CG, Mohamed S, Kaur K, Sulaiman AH, Zainal NZ, Taib NA, MyBCC Study group (2017). Perceived distress and its association with depression and anxiety in breast cancer patients. PLoS One.

[33] Montgomery M, McCrone SH (2010). Psychological distress associated with the diagnostic phase for suspected breast cancer: systematic review. J Adv Nurs.

[34] Saboonchi F, Petersson LM, Wennman-Larsen A, Alexanderson K, Brännström R, Vaez M (2014). Changes in caseness of anxiety and depression in breast cancer patients during the first year following surgery: patterns of transiency and severity of the distress response. Eur J Oncol Nurs.

[35] Golden-Kreutz DM, Andersen BL (2004). Depressive symptoms after breast cancer surgery: relationships with global, cancer-related, and life event stress. Psychooncology.

[36] Boing L, Pereira GS, Araújo CDCR, Sperandio FF, Loch MDSG, Bergmann A (2019). Factors associated with depression symptoms in women after breast cancer. Rev Saude Publica.

[37] Rogers LQ, Courneya KS, Anton PM, Verhulst S, Vicari SK, Robbs RS (2017). Effects of a multicomponent physical activity behavior change intervention on fatigue, anxiety, and depressive symptomatology in breast cancer survivors: randomized trial. Psychooncology.

[38] Luecken LJ, Compas BE (2002). Stress, coping, and immune function in breast cancer. Ann Behav Med.

[39] Di Martino MT, Riillo C, Scionti F, Grillone K, Polerà N, Caracciolo D (2021). miRNAs and lncRNAs as Novel Therapeutic Targets to Improve Cancer Immunotherapy. Cancers (Basel).

[40] Karakoyun-Celik O, Gorken I, Sahin S, Orcin E, Alanyali H, Kinay M (2010). Depression and anxiety levels in woman under follow-up for breast cancer: relationship to coping with cancer and quality of life. Med Oncol.

[41] Stanton AL, Danoff-Burg S, Cameron CL, Bishop M, Collins CA, Kirk SB (2000). Emotionally expressive coping predicts psychological and physical adjustment to breast cancer. J Consult Clin Psychol.

[42] Osowiecki DM, Compas BE (1999). A prospective study of coping, perceived control, and psychological adaptation to breast cancer. Cogn Ther Res.

[43] Skeels MM, Unruh KT, Powell C, Pratt W (2010). Catalyzing Social Support for Breast Cancer Patients. Proc SIGCHI Conf Hum Factor Comput Syst.

[44] Holland KD, Holahan CK (2003). The relation of social support and coping to positive adaptation to breast cancer. Psychol Health.

[45] Pinto AC, de Azambuja E (2011). Improving quality of life after breast cancer: dealing with symptoms. Maturitas.

[46] Turner-Cobb JM, Sephton SE, Koopman C, Blake-Mortimer J, Spiegel D (2000). Social support and salivary cortisol in women with metastatic breast cancer. Psychosom Med.

[47] Feig SA (1984). Mammographic screening for breast cancer. N Engl J Med.

[48] Tabár L, Fagerberg CJ, Gad A, Baldetorp L, Holmberg LH, Gröntoft O (1985). Reduction in mortality from breast cancer after mass screening with mammography. Randomised trial from the Breast Cancer Screening Working Group of the Swedish National Board of Health and Welfare. Lancet.

[49] Gotzsche PC, Jorgensen KJ (2013). Screening for breast cancer with mammography. Cochrane Database Syst Rev.

[50] Moss SM, Wale C, Smith R, Evans A, Cuckle H, Duffy SW, Trial Management Group (2015). Effect of mammographic screening from age 40 years on breast cancer mortality in the UK Age trial at 17 years' follow-up: a randomised controlled trial. Lancet Oncol.

[51] Shapiro S, Strax P, Venet L (1971). Periodic breast cancer screening in reducing mortality from breast cancer. JAMA.

[52] Obeagu EI, Obeagu GU (2024). Exploring the profound link: Breastfeeding’s impact on alleviating the burden of breast cancer–A review. Medicine (Baltimore).

[53] Polednak AP (2002). Trends in, and predictors of, breast-conserving surgery and radiotherapy for breast cancer in Connecticut 1988-1997. Int J Radiat Oncol Biol Phys.

[54] Christiansen P, Mele M, Bodilsen A, Rocco N, Zachariae R (2022). Breast-Conserving Surgery or Mastectomy?: Impact on Survival. Ann Surg Open.

[55] De la Cruz Ku G, Karamchandani M, Chambergo-Michilot D, Narvaez-Rojas AR, Jonczyk M, Príncipe-Meneses FS (2022). Does Breast-Conserving Surgery with Radiotherapy have a Better Survival than Mastectomy? A Meta-Analysis of More than 1,500,000 Patients. Ann Surg Oncol.

[56] Tuttle TM, Abbott A, Arrington A, Rueth N (2010). The increasing use of prophylactic mastectomy in the prevention of breast cancer. Curr Oncol Rep.

[57] Coopey SB (2023). Contralateral Prophylactic Mastectomy in Average Risk Women: Who Can Choose This Wisely?. Ann Surg Oncol.

[58] van Maaren MC, de Munck L, de Bock GH, Jobsen JJ, van Dalen T, Linn SC (2016). 10 year survival after breast-conserving surgery plus radiotherapy compared with mastectomy in early breast cancer in the Netherlands: a population-based study. Lancet Oncol.

[59] Lee ES, Lee MK, Kim SH, Ro JS, Kang HS, Kim SW (2011). Health-related quality of life in survivors with breast cancer 1 year after diagnosis compared with the general population: a prospective cohort study. Ann Surg.

[60] Ganz PA, Kwan L, Stanton AL, Bower JE, Belin TR (2011). Physical and psychosocial recovery in the year after primary treatment of breast cancer. J Clin Oncol.

[61] Thewes B, Butow P, Girgis A, Pendlebury S (2004). The psychosocial needs of breast cancer survivors; a qualitative study of the shared and unique needs of younger versus older survivors. Psychooncology.

[62] Ganz PA, Schag AC, Lee JJ, Polinsky ML, Tan SJ (1992). Breast conservation versus mastectomy. Is there a difference in psychological adjustment or quality of life in the year after surgery?. Cancer.

[63] Rosenberg SM, Tracy MS, Meyer ME, Sepucha K, Gelber S, Hirshfield-Bartek J (2013). Perceptions, knowledge, and satisfaction with contralateral prophylactic mastectomy among young women with breast cancer: a cross-sectional survey. Ann Intern Med.

[64] Lazovich D, Solomon CC, Thomas DB, Moe RE, White E (1999). Breast conservation therapy in the United States following the 1990 National Institutes of Health Consensus Development Conference on the treatment of patients with early stage invasive breast carcinoma. Cancer.

[65] Rowland JH, Desmond KA, Meyerowitz BE, Belin TR, Wyatt GE, Ganz PA (2000). Role of breast reconstructive surgery in physical and emotional outcomes among breast cancer survivors. J Natl Cancer Inst.

[66] Burgess C, Cornelius V, Love S, Graham J, Richards M (2005). Depression and anxiety in women with early breast cancer: five year observational cohort study. BMJ.

[67] Hamer J, McDonald R, Zhang L, Verma S, Leahey A, Ecclestone C (2017). Quality of life (QOL) and symptom burden (SB) in patients with breast cancer. Support Care Cancer.

[68] St Fleur RG, St George SM, Feaster DJ, Lee TK, Antoni MH (2023). Functions of Resiliency Traits and Processes in Differential Effects of CBSM on Quality of Life in Breast Cancer Survivors: A Moderated Mediation Model. Int J Behav Med.

[69] Faroughi F, Fathnezhad-Kazemi A, Sarbakhsh P (2023). Factors affecting quality of life in women with breast cancer: a path analysis. BMC Womens Health.

[70] Yan J, Wu C, He C, Lin Y, He S, Du Y (2022). The social support, psychological resilience and quality of life of nurses in infectious disease departments in China: A mediated model. J Nurs Manag.

[71] Wefel JS, Vardy J, Ahles T, Schagen SB (2011). International Cognition and Cancer Task Force recommendations to harmonise studies of cognitive function in patients with cancer. Lancet Oncol.

[72] Ahles TA, Root JC, Ryan EL (2012). Cancer-and cancer treatment-associated cognitive change: an update on the state of the science. J Clin Oncol.

[73] Bender CM, Ergÿn FS, Rosenzweig MQ, Cohen SM, Sereika SM (2005). Symptom clusters in breast cancer across 3 phases of the disease. Cancer Nurs.

[74] Henselmans I, Helgeson VS, Seltman H, de Vries J, Sanderman R, Ranchor AV (2010). Identification and prediction of distress trajectories in the first year after a breast cancer diagnosis. Health Psychol.

[75] Trace K, Northouse L, Kritpracha C, Schafenacker A, Mood D (2004). Coping strategies and quality of life in women with advanced breast cancer and their family caregivers. Psychol Health.

[76] Hooning MJ, Botma A, Aleman BM, Baaijens MH, Bartelink H, Klijn JG (2007). Long-term risk of cardiovascular disease in 10-year survivors of breast cancer. J Natl Cancer Inst.

[77] Coleman RE, Banks LM, Girgis SI, Kilburn LS, Vrdoljak E, Fox J, Intergroup Exemestane Study group (2007). Skeletal effects of exemestane on bone-mineral density bone biomarkers, and fracture incidence in postmenopausal women with early breast cancer participating in the Intergroup Exemestane Study (IES): a randomised controlled study. Lancet Oncol.

[78] Sharma N, Purkayastha A (2017). Factors Affecting Quality of Life in Breast Cancer Patients: A Descriptive and Cross-sectional Study with Review of Literature. J Midlife Health.

[79] Halkett GK, Kristjanson LJ, Lobb E, O'Driscoll C, Taylor M, Spry N (2010). Meeting breast cancer patients’ information needs during radiotherapy: what can we do to improve the information and support that is currently provided?. Eur J Cancer Care (Engl).

[80] Schover LR, Yetman RJ, Tuason LJ, Meisler E, Esselstyn CB, Hermann RE (1995). Partial mastectomy and breast reconstruction. A comparison of their effects on psychosocial adjustment body image and sexuality. Cancer.

[81] Ussher JM, Perz J, Gilbert E (2012). Sexuality after breast cancer treatment. Cancer Nurs.

